# Bioavailable heavy-metal pressure is linked to microbial functional vulnerability and carbon-use niche contraction in karst agricultural soils

**DOI:** 10.3389/fmicb.2026.1887715

**Published:** 2026-07-15

**Authors:** Wenming Liu, Jiaxin Shen, Yongxu Lin, Long Zhou, Jiaqi Wang, Jie Liu, Xujin Wei

**Affiliations:** 1 Endoscopic Center, The First Affiliated Hospital, Fujian Medical University, Fuzhou, Fujian, China; 2 Endoscopic Center, National Regional Medical Center, Binhai Campus of the First Affiliated Hospital, Fujian Medical University, Fuzhou, Fujian, China; 3 College of Chemistry & Materials Science, Longyan University, Longyan, Fujian, China; 4 Digestive Endoscopy Center, Fuzhou University Affiliated Provincial Hospital, Fuzhou, Fujian, China

**Keywords:** bioavailable metals, carbon cycling, carbon-source utilization, karst soil, microbial functional vulnerability

## Abstract

Heavy-metal contamination threatens microbial processes in karst agricultural soils, but functional responses to the bioavailable metal fraction remain insufficiently resolved. We integrated soil properties, total and DTPA-extractable metals, microbial abundance and community profiles, and Biolog EcoPlate traits to quantify microbial functional vulnerability in Pb–Zn mining-affected agricultural soils. DTPA-extractable metal pressure was positively associated with functional vulnerability (Spearman *ρ* = 0.626, *p* < 0.001) and negatively associated with carbon-use niche breadth (*ρ* = −0.481, *p* < 0.001). Contraction occurred across several substrate classes and remained consistent in covariate-adjusted and sensitivity analyses. Community reorganization, independent contaminated-soil datasets, and a targeted qPCR microcosm experiment further connected the field pattern with shifts in microbial community structure and functional marker genes. These findings identify carbon-use niche contraction as an informative functional response to bioavailable metal mixtures and support its use alongside chemical indicators when assessing remediation and sustainable management of contaminated karst soils.

## Introduction

Karst agricultural soils are ecologically sensitive systems because thin soil layers, carbonate parent materials, high permeability, and close hydrological connectivity can amplify the consequences of soil degradation (Xu et al., 2025). Carbonate bedrock, rapid weathering, and Ca- and Mg-rich geochemistry jointly shape soil organic carbon, nitrogen availability, and microbial activity in karst landscapes (Xiao et al., 2017; Lin et al., 2026). In mining-affected karst regions, agricultural soils may accumulate Pb, Zn, Cd, Cu, and other metals from tailings, smelting, irrigation, dust deposition, and legacy contamination. Multiple-metal contamination has also been shown to alter soil microbial communities in karst tea plantations (Huang et al., 2023). Mining-derived tailings can contribute substantially to topsoil organic matter pools in adjacent farmlands, complicating the relationship between metal inputs and soil carbon dynamics (Zhang et al., 2023). The accumulated metals can threaten crop production, soil fertility, microbial processes, and downstream exposure pathways. However, total metal concentration alone does not necessarily represent the fraction that interacts with microbial cells or participates in near-term biogeochemical disturbance.

Bioavailable metals are therefore central to understanding microbial responses in contaminated soils. DTPA-extractable metal fractions are commonly used as operational indicators of plant- and microbe-accessible metal pools. In karst agricultural soils, where pH, carbonate chemistry, organic matter, and cation exchange capacity can jointly regulate metal mobility, DTPA-extractable metals may capture microbial exposure more closely than total metal burden. Field evidence indicates that DTPA-extractable Cu, rather than total Cu, can be the dominant factor explaining variation in microbial biomass and carbon mineralization in mining-affected soils (Peng et al., 2024). A microbial community may appear taxonomically resilient under a total contamination gradient while still experiencing functional stress along the bioavailable fraction.

Soil microorganisms mediate carbon, nitrogen, phosphorus, and sulfur cycling, making their functional capacity central to soil productivity and ecological recovery. In this study, microbial functional vulnerability denotes the co-occurrence of high bioavailable metal pressure with reduced carbon-use breadth, diversity, and biomass-normalized activity. The concept complements taxonomic diversity by quantifying functional condition directly; it also differs from resistance and resilience, which describe responses during and after a defined disturbance and therefore require temporal measurements. Evidence from mining-affected karst soils shows that metal stress can reorganize microbial communities and functional genes involved in carbon and nitrogen cycling (Peng et al., 2025; Wang et al., 2025). However, most assessments still treat exposure, community composition, and individual functional endpoints separately. Integrating these dimensions provides a way to detect functional deterioration that may remain inconspicuous when evaluation relies on taxonomic diversity alone.

Biolog EcoPlate carbon-source utilization data offer a useful functional phenotype for this question. Although EcoPlate assays do not represent the full *in situ* metabolic repertoire, community-level sole-carbon-source utilization profiles provide sample-level measures of carbon-use richness, intensity, substrate-group preference, and functional ordination (Garland and Mills, 1991). EcoPlate-based approaches have been used to detect pollutant-related shifts in microbial carbon metabolism, with contaminated soils typically showing altered substrate richness and functional diversity (Tao et al., 2023). When paired with geochemical measurements and microbial community profiles, these traits can be reinterpreted as carbon-use niche breadth and functional contraction under metal stress.

Because EcoPlate profiles are generated under standardized incubation conditions, they are used here to compare assay-detectable carbon-use potential among samples rather than to quantify actual *in situ* carbon fluxes. This distinction is important for avoiding overinterpretation of carbon-use niche contraction as a direct measure of field metabolism.

Here, we tested whether DTPA-extractable metal mixtures are associated with carbon-use niche contraction and an integrated microbial functional-vulnerability phenotype in Pb–Zn mining-affected karst agricultural soils. The framework advances conventional diversity-based assessment in two respects. First, carbon-use niche breadth represents the range and evenness of assay-detectable substrate use, thereby translating community activity into an ecologically interpretable functional phenotype. Second, the functional-vulnerability and metal-specific function-loss indices combine exposure and response variables in transparent, directionally aligned metrics that can be reproduced across samples and compared with community-level indicators. We evaluated these relationships using covariate-adjusted, robustness, community-ordination, path-structured association, and feature-prioritization analyses, with independent datasets and a qPCR microcosm experiment used to examine the broader biological consistency of the field pattern.

## Materials and methods

### Study design and data sources

This analysis used sample-level karst agricultural soil data containing soil physicochemical variables, total heavy metals, DTPA-extractable metals, microbial abundance measurements, bacterial/fungal community information, and Biolog EcoPlate carbon-source utilization measurements from Li et al. (2018). The study asked a systems-level question: whether bioavailable metal pressure can be integrated with microbial functional phenotypes to define a quantitative functional-vulnerability state.

The discovery dataset included 44 samples with complete soil and Biolog EcoPlate data, spanning uncontaminated reference soils and Pb–Zn mining-affected karst agricultural soils. The original sampling design included 4 CK reference soils, 13 paddy-field soils, 13 corn-field soils, and 14 citrus-field soils, with sequencing data reported under BioProject PRJNA418926. Available variables included pH, soil organic carbon (SOC), cation exchange capacity (CEC), microbial biomass-related measurements, total and DTPA-extractable Pb, Zn, Cu, and Cd, bacterial/fungal abundance indicators, OTU-level community profiles, and Biolog EcoPlate readings for 31 carbon sources. All variables were harmonized at the sample level before index construction and statistical analysis.

### Metal exposure indices

Metal exposure was represented using several complementary indices. The primary exposure was a DTPA-extractable multi-metal pressure index constructed from bioavailable Pb, Zn, Cu, and Cd. Additional indices included a total metal index, a DTPA metal mixture principal component, toxicity-weighted DTPA mixture scores, local CK-referenced contamination factors, local geo-accumulation indices, potential ecological risk indices, and DTPA/total bioavailability ratios. These indices were used as comparative exposure axes rather than formal regulatory classifications.

Because Pb, Zn, Cu, and Cd co-occurred in the contaminated soils, mixture-level interpretation was prioritized. Variance inflation and single-metal dominance analyses were used to determine whether individual metals could be separated statistically from the broader co-contamination gradient. Individual metal coefficients were interpreted cautiously when collinearity was present.

For the primary multi-metal indices, metal concentrations were log-transformed as log1p(x), standardized across samples, and averaged across metals. The total metal index was the mean standardized log1*p* value of total Pb, Zn, Cu, and Cd. The DTPA metal index was the mean standardized log1*p* value of DTPA-extractable Pb, Zn, Cu, and Cd. The DTPA mixture PC1 was obtained from the standardized DTPA metal matrix. Local contamination factors were calculated relative to the CK group mean, geo-accumulation indices used the same local CK reference with the conventional 1.5 correction factor, and potential ecological risk indices used common toxic-response weights of Cd = 30, Pb = 5, Cu = 5, and Zn = 1. These risk scores were used only as comparative geochemical indicators.

### Carbon-source utilization and carbon-use niche metrics

Biolog EcoPlate measurements were used to derive carbon-use intensity, carbon-source richness, Shannon diversity, and carbon-use niche breadth. Carbon sources were grouped into ecologically interpretable substrate classes, including amino acids, polymers, carbohydrates, carboxylic acids, polyols/phosphates, amino sugars, and amines. Group-level responses were analyzed to determine whether metal-associated functional contraction was broad or restricted to isolated substrates.

Carbon-use distance from the uncontaminated reference centroid was calculated to describe functional deviation from the CK-like state. Carbon-use per SOC was used as an index of microbial carbon processing relative to the available soil carbon pool.

Carbon-use richness was calculated as the number of EcoPlate carbon sources with response values greater than 0.25, with a second richness count greater than zero retained as a sensitivity descriptor. Carbon-use Shannon diversity and evenness were calculated from the 31 carbon-source response profile. Carbon-use niche breadth was defined as the mean of standardized carbon-source richness, standardized Shannon diversity, and standardized evenness; higher values indicate broader assay-detectable carbon-use capacity, whereas lower values indicate contraction of the community-level carbon-use phenotype. Carbon-use per SOC was calculated as total carbon-source utilization divided by SOC and was interpreted as assay-based carbon-use potential relative to the available soil carbon pool.

### Functional vulnerability and metal-specific function loss

The microbial functional-vulnerability index integrated five directionally aligned standardized components: DTPA metal pressure, loss of carbon-source richness, loss of carbon-use Shannon diversity, lower carbon use relative to SOC, and lower MBC relative to SOC. Accordingly, a higher value denotes stronger bioavailable metal pressure together with a narrower and less active carbon-use phenotype. The metal-specific function-loss index was calculated as standardized DTPA metal pressure minus carbon-use niche breadth; higher values denote a larger imbalance between metal exposure and retained carbon-use capacity. These indices summarize complementary ecological dimensions: the former captures the overall exposure-response state, whereas the latter focuses on functional contraction along the metal-pressure gradient.

Sensitivity analyses evaluated whether the main association was dependent on any single component of the vulnerability index. Variants included leave-one-component-out indices and winsorized versions of the vulnerability and function-loss scores.

The functional-vulnerability index was calculated as [z(DTPA metal index) − z(carbon-source richness) − z(carbon-use Shannon diversity) − z(carbon use/SOC) − z(MBC/SOC)]/5. Carbon-use niche breadth was the mean of standardized richness, Shannon diversity, and evenness, and metal-specific function loss was z(DTPA metal index) − carbon-use niche breadth. The sign of each component was oriented before averaging so that larger values consistently represented greater vulnerability or function loss. Sensitivity analyses evaluated equal-weight, leave-one-component-out, and winsorized versions of the indices.

### Community-function coupling and OTU-level analyses

OTU-level bacterial community data were integrated with carbon-use ordination to test whether microbial community structure was coupled to carbon-use functional structure. Indicator OTU analysis was used to identify taxa associated with increasing or declining metal-pressure regions. Exploratory threshold scans were used to examine whether carbon-use endpoints and indicator taxa showed nonlinear changes along the DTPA metal gradient.

Co-occurrence network analysis was performed as an exploratory community-context layer. OTUs were selected by prevalence and relative abundance, transformed using centered log-ratio procedures, and correlated within low- and high-DTPA groups. Network topology and network-core scores were compared between low- and high-metal-pressure samples. These networks were interpreted as co-occurrence structures, not proof of direct ecological interactions.

### Statistical analysis

Spearman correlation was used for monotonic sample-level associations. Covariate-adjusted linear models evaluated associations between metal exposure indices and functional endpoints after adjustment for pH, SOC, and CEC. False discovery rate correction was applied where multiple related tests were performed. Block-wise dominance analysis estimated the relative explanatory contribution of bioavailable metals, total metal risk, soil properties, microbial abundance, and single-metal bioavailability. PERMANOVA was used for external Bray-Curtis community separation analysis following the non-parametric multivariate framework of Anderson (2001).

Feature prioritization, threshold scanning, and path-structured association modeling were used to integrate the principal exposure, community, and functional variables. Random-forest permutation importance ranked candidate predictors, with repeated cross-validation used to assess ranking stability. Threshold scans located response regions along the DTPA gradient. The path-structured model estimated standardized associations among metal-mixture pressure, community/network scores, carbon-use niche breadth, and functional vulnerability while adjusting for soil covariates. Given the cross-sectional discovery design, model paths were interpreted as multivariable associations.

### Independent contaminated-soil support

Independent contaminated-soil evidence was used as contextual support rather than as a one-to-one validation of the discovery dataset. Published table-level functional matrices from contaminated agricultural soil studies were inspected for microbial biomass, nutrient-cycle, CAZy, and NCyc evidence, including a long-term Hg-contaminated soil metagenome with CAZy and NCyc summaries (Frey et al., 2022) and a heavy-metal-contaminated soil nitrogen-cycling metagenomic matrix under fertilization regimes (Shi et al., 2023). A lightweight analysis of 16 raw 16S samples from an independent contaminated agricultural soil dataset reported by Li et al. (2024) was also performed using R1 exact-sequence summaries to assess whether contaminated and uncontaminated soils showed community-structure separation. Because this raw-data analysis did not use full denoising, paired-end merging, or complete taxonomic assignment, it was treated as supportive community evidence only.

### Biological validation microcosm qPCR

To provide targeted biological validation, a controlled soil microcosm qPCR assay was designed to test whether mixed bioavailable heavy-metal pressure was accompanied by marker-gene changes in the same functional direction inferred from the field data. Homogenized low-contamination agricultural soil was passed through a 2-mm sieve and allocated into sterile incubation vessels equivalent to 50 g dry soil. Three treatments were prepared with five biological replicates each: an unamended control, a medium mixed-metal amendment, and a high mixed-metal amendment. The medium treatment received Pb, Zn, Cu, and Cd at 300, 500, 100, and 3 mg kg^−1^ dry soil, respectively, whereas the high treatment received 900, 1,500, 300, and 9 mg kg^−1^ dry soil, respectively. Microcosms were adjusted to 60% water-holding capacity, loosely capped, incubated at 25 degrees C in the dark, and maintained gravimetrically.

At the experimental endpoint, soil DNA was extracted using a commercial soil DNA kit with bead-beating disruption. DTPA-extractable Pb, Zn, Cu, and Cd were measured, where material allowed, to confirm the bioavailable metal-pressure gradient. SYBR Green qPCR was used to quantify bacterial 16S rRNA genes, nifH, bacterial amoA, and czcA. Standard curves were generated from serially diluted plasmid standards, synthetic fragments, or purified target amplicons. Accepted runs required amplification efficiency of approximately 90–110%, *R*^2^ > = 0.99, negative controls below the quantification limit, and a single dominant melt-curve product. Gene abundance was expressed as log10 copies g^−1^ dry soil and as log10 (target/16S) for functional and resistance markers.

### Reproducibility

All analyses were performed using local scripts on a desktop workstation. Source files, intermediate CSV tables, figures, Markdown reports, and an embedded HTML manuscript package were retained to preserve traceability.

## Results

### Bioavailable metal mixtures defined the exposure gradient linked to vulnerability

**Figure 1:**
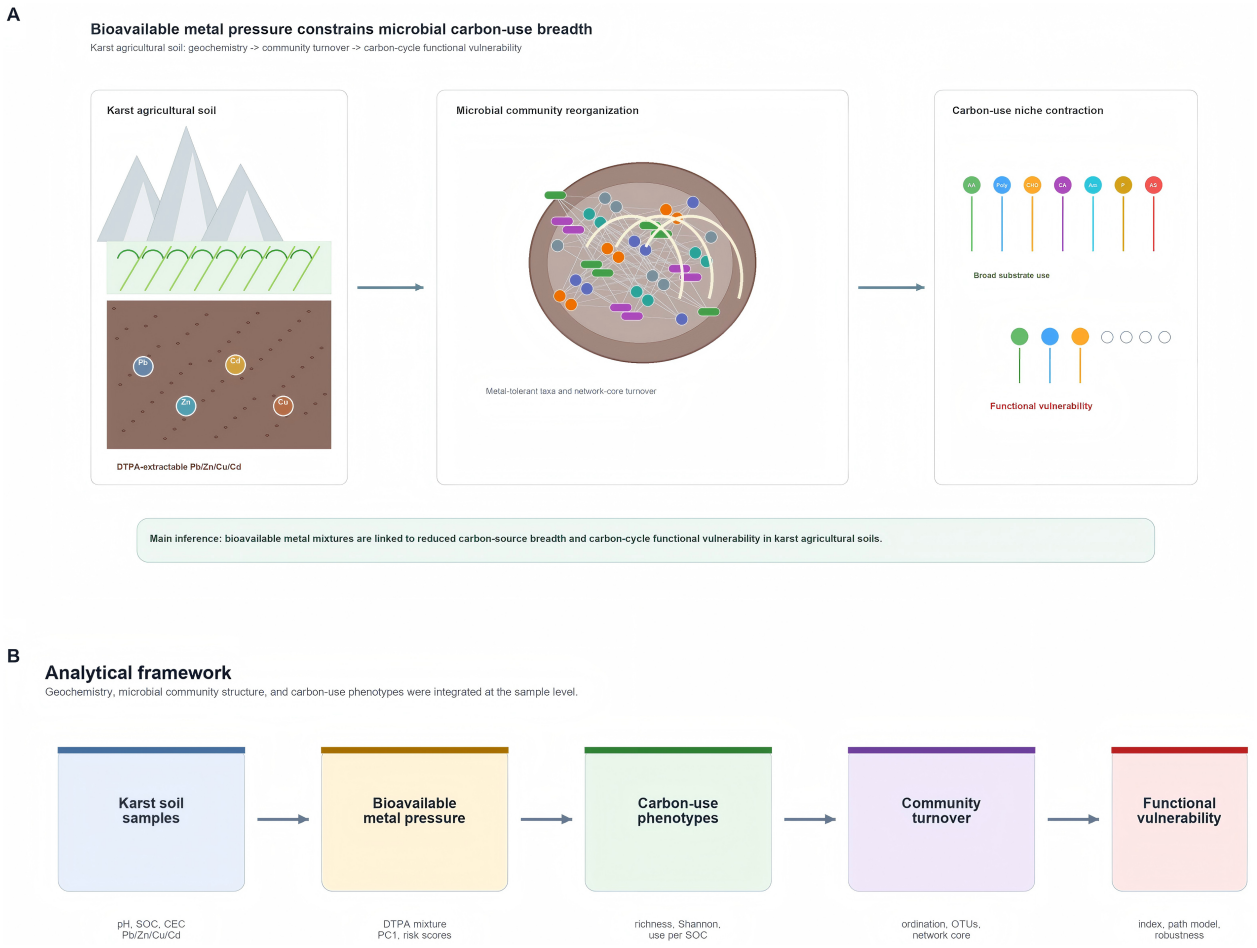
Conceptual framework and study design. **(A)** Conceptual framework linking DTPA-extractable Pb, Zn, Cu, and Cd with microbial community reorganization, carbon-use niche contraction, and functional vulnerability. **(B)** Study workflow integrating soil properties, metal fractions, Biolog EcoPlate profiles, microbial community features, independent datasets, and the qPCR microcosm experiment. DTPA, diethylenetriaminepentaacetic acid; FVI, functional-vulnerability index.

DTPA-extractable Pb, Zn, Cu, and Cd formed a coherent exposure gradient across the study soils (Figures 1A,B). This gradient provided the common geochemical axis for evaluating microbial community and carbon-use responses.

**Figure 2:**
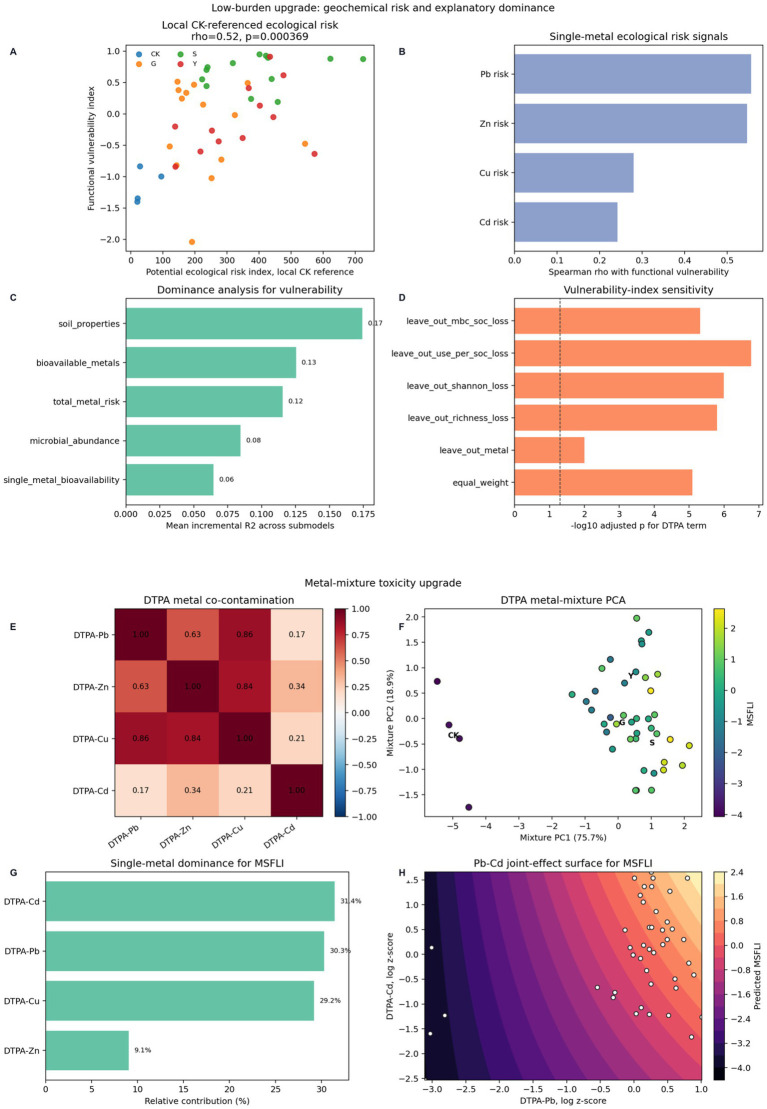
Geochemistry and functional vulnerability. **(A–D)** CK-referenced ecological risk, single-metal contributions, explanatory blocks, and index sensitivity. **(E–H)** Correlations among DTPA-extractable metals, mixture principal-component analysis, metal-specific contributions, and the Pb–Cd joint-response surface. Colors denote metal identity or exposure level as indicated in each panel. The coordinated mixture pattern identifies DTPA-extractable co-contamination as the principal exposure gradient associated with functional vulnerability.

CK-referenced ecological risk was positively associated with the functional-vulnerability index (Spearman *ρ* = 0.52, *p* = 3.69 × 10^−4^; Figure 2A), indicating that the geochemical contrast aligned with microbial functional impairment. Single-metal ecological-risk signals were strongest for Pb and Zn, with weaker but positive Cu and Cd signals (Figure 2B). Block-wise dominance analysis showed that soil properties, bioavailable metals, and total-metal risk each explained part of the vulnerability pattern, with bioavailable metals retained as a major explanatory block rather than a redundant chemical descriptor (Figure 2C). The vulnerability index was also robust to alternative component definitions and weighting schemes, supporting the use of a composite phenotype rather than a single assay readout (Figure 2D; Supplementary Figure 1).

The same exposure landscape was clearly mixture-structured. DTPA-Pb, DTPA-Zn, and DTPA-Cu were strongly correlated, whereas DTPA-Cd was less tightly coupled to the other metals (Figure 2E). The first DTPA mixture principal component explained 75.7% of mixture variance and separated the low-pressure CK samples from contaminated agricultural soils (Figure 2F). For the metal-specific function-loss index, relative single-metal contributions were highest for DTPA-Cd, DTPA-Pb, and DTPA-Cu, with a smaller contribution from DTPA-Zn (Figure 2G). A Pb–Cd joint-effect surface further showed higher functional loss across the combined metal-pressure space (Figure 2H). Together, these results support the interpretation that bioavailable co-contamination, rather than any one metal alone, provided the most appropriate exposure axis for subsequent functional analyses.

In ecological terms, this section establishes that DTPA-extractable metal mixtures provide the exposure gradient against which microbial functional responses can be interpreted.

### Carbon-use niche breadth was the main functional phenotype of metal-associated vulnerability

DTPA metal pressure was negatively associated with carbon-source richness, total carbon-use intensity, carbon use relative to SOC, Shannon diversity, and the composite carbon-use niche-breadth score (all adjusted *p* ≤ 0.005). In contrast, DTPA metal pressure was positively associated with functional vulnerability (*ρ* = 0.626, adjusted *p* < 0.001) and metal-specific function loss (*ρ* = 0.734, adjusted *p* < 0.001). These coordinated responses identify broad carbon-use niche contraction, rather than loss of a single substrate response, as the dominant functional pattern along the bioavailable metal gradient.

**Figure 3:**
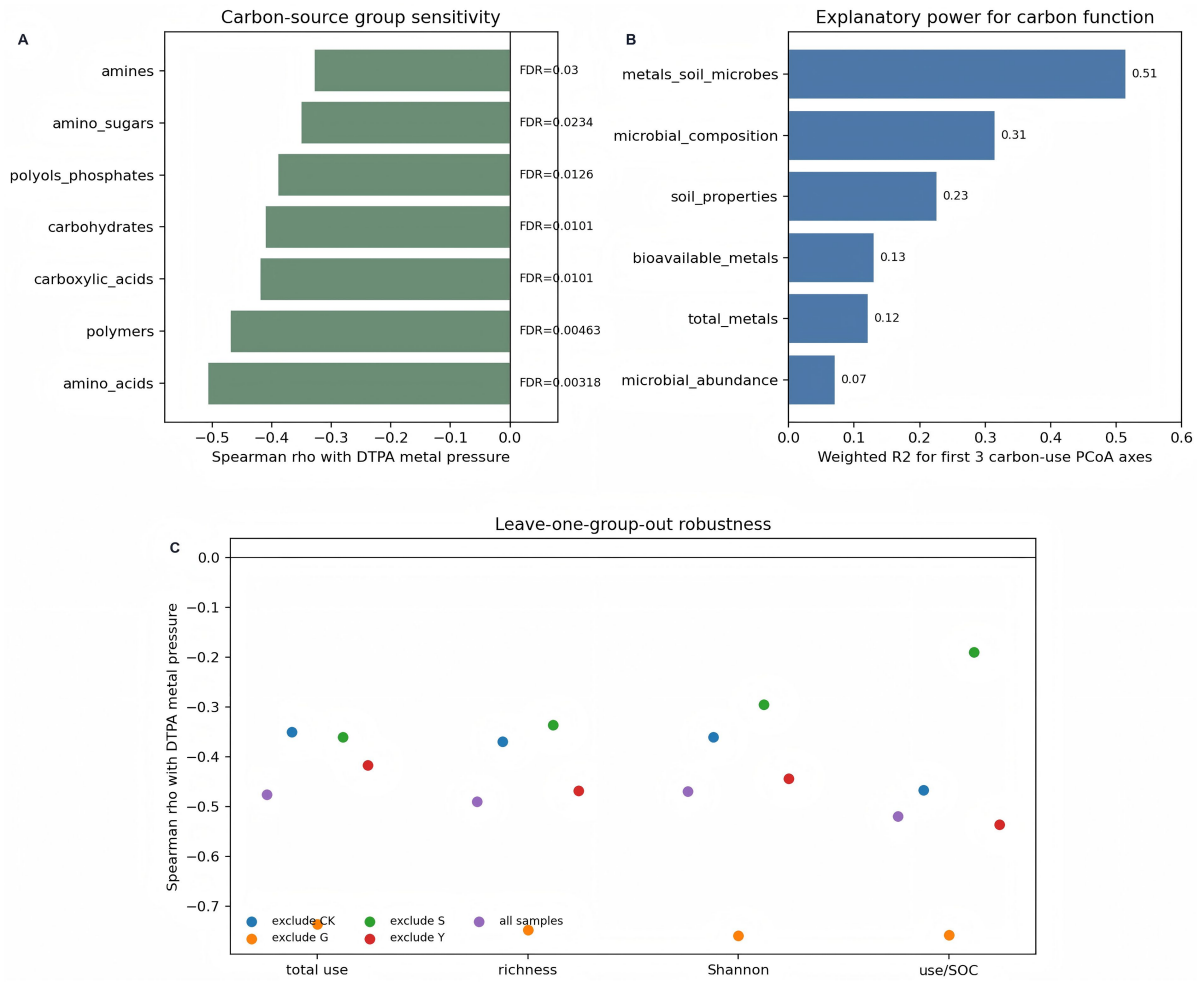
Carbon-source niche contraction. **(A)** Spearman associations between DTPA metal pressure and seven EcoPlate substrate groups; labels show false-discovery-rate-adjusted *p*-values. **(B)** Weighted explanatory power for the first three carbon-use principal-coordinate axes. **(C)** Leave-one-group-out associations for total carbon use, richness, Shannon diversity, and carbon use relative to soil organic carbon. Point colors identify the omitted soil group; purple denotes the complete dataset. Negative associations across substrate groups demonstrate broad carbon-use niche contraction along the DTPA gradient.

Carbon-source group analysis showed that this was a broad niche-narrowing signal rather than a response of one substrate class. All seven substrate groups were negatively associated with DTPA metal pressure after false-discovery correction: amino acids (*ρ* = −0.506, FDR = 0.00318), polymers (*ρ* = −0.469, FDR = 0.00464), carboxylic acids (*ρ* = −0.418, FDR = 0.0101), carbohydrates (*ρ* = −0.410, FDR = 0.0101), polyols/phosphates (*ρ* = −0.389, FDR = 0.0126), amino sugars (*ρ* = −0.350, FDR = 0.0234), and amines (*ρ* = −0.328, FDR = 0.0300; Figure 3A). Explanatory-power analysis placed metal, soil, and microbial blocks within the same carbon-use ordination framework, indicating that carbon-use profiles captured an integrated functional response rather than only one derived index (Figure 3B).

Robustness analysis supported this interpretation. Leave-one-component-out and winsorized versions of the vulnerability endpoint remained positively associated with DTPA pressure; for example, the equal-weight vulnerability index showed *ρ* = 0.621 (*p* = 6.743 × 10^–6^), and the winsorized version showed *ρ* = 0.626 (*p* = 7.04 × 10^–6^). Winsorized carbon-use niche breadth remained negatively associated with DTPA pressure (*ρ* = −0.481, *p* = 9.41 × 10^–4^). Leave-one-group-out analyses further indicated that the metal-carbon association did not depend on a single soil group (Figure 3C; Supplementary Figure 2). Additional phenotype–genotype context for carbohydrate- and polymer-related carbon processing is provided in Supplementary Figure 3, but it is used only as functional interpretation support. Overall, these findings indicate that the main biological signal was a broad carbon-use niche contraction associated with bioavailable metal pressure.

### Community reorganization accompanied carbon-use contraction

**Figure 4:**
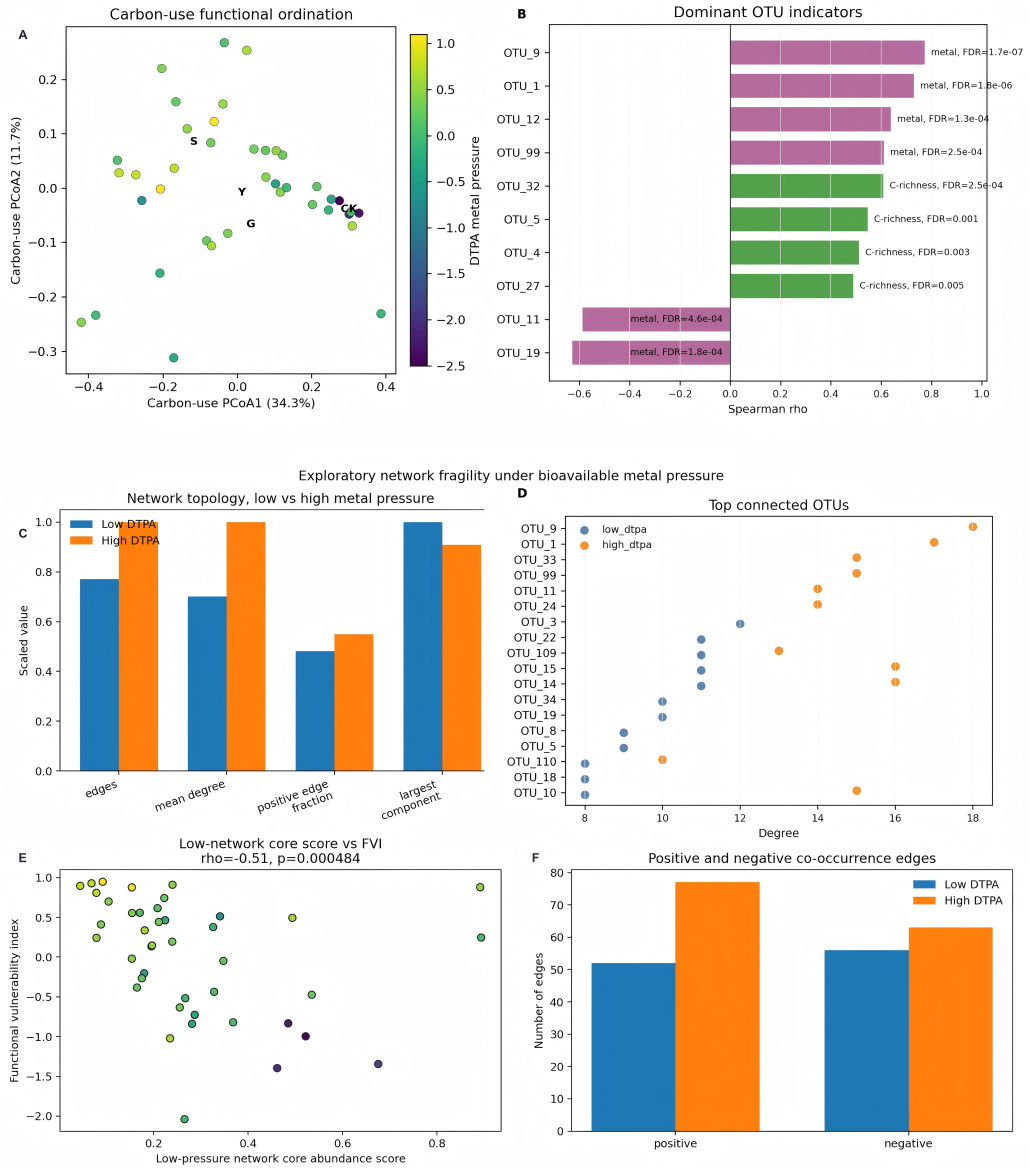
Community turnover and network-core shifts. **(A)** Carbon-use functional ordination colored by DTPA metal pressure. **(B)** Dominant operational taxonomic unit (OTU) indicators associated with metal pressure (purple) or carbon-use richness (green). **(C, D)** Network topology and highly connected OTUs under low (blue) and high (orange) DTPA pressure. **(E)** Low-pressure network-core abundance versus functional vulnerability; points are colored by DTPA pressure. **(F)** Positive and negative co-occurrence edges. Community composition and network-core structure changed systematically across the metal-pressure gradient.

The functional phenotype was then evaluated in relation to bacterial community structure. Carbon-use ordination and microbial community ordination showed evidence of coupling: a Procrustes comparison between genus-level ordination and carbon-use ordination was significant (statistic = 0.839, *p* = 0.004; *n* = 39), while Mantel tests were directionally positive but weaker for genus-level profiles (*r* = 0.160, *p* = 0.093) and dominant OTU profiles (*r* = 0.142, *p* = 0.142; Figure 4A). Indicator OTU analysis identified taxa associated with increasing or declining regions of the metal-pressure and carbon-richness gradients (Figure 4B).

Network topology and connected OTUs differed between low- and high-DTPA soils (Figures 4C,D). Low-pressure network-core scores were negatively associated with functional vulnerability and metal-specific function loss, whereas high-pressure network-core scores were positively associated with functional vulnerability (FDR < 0.01; Figure 4E). Indicator scans also located response regions along the DTPA gradient (Figure 4F). Together, the community analyses show that carbon-use contraction coincided with systematic turnover in community composition and network-core structure.

The concordant ordination, indicator, and network patterns place the functional response within a reproducible community-reorganization gradient.

### Integrated models prioritized the metal-carbon vulnerability link

**Figure 5:**
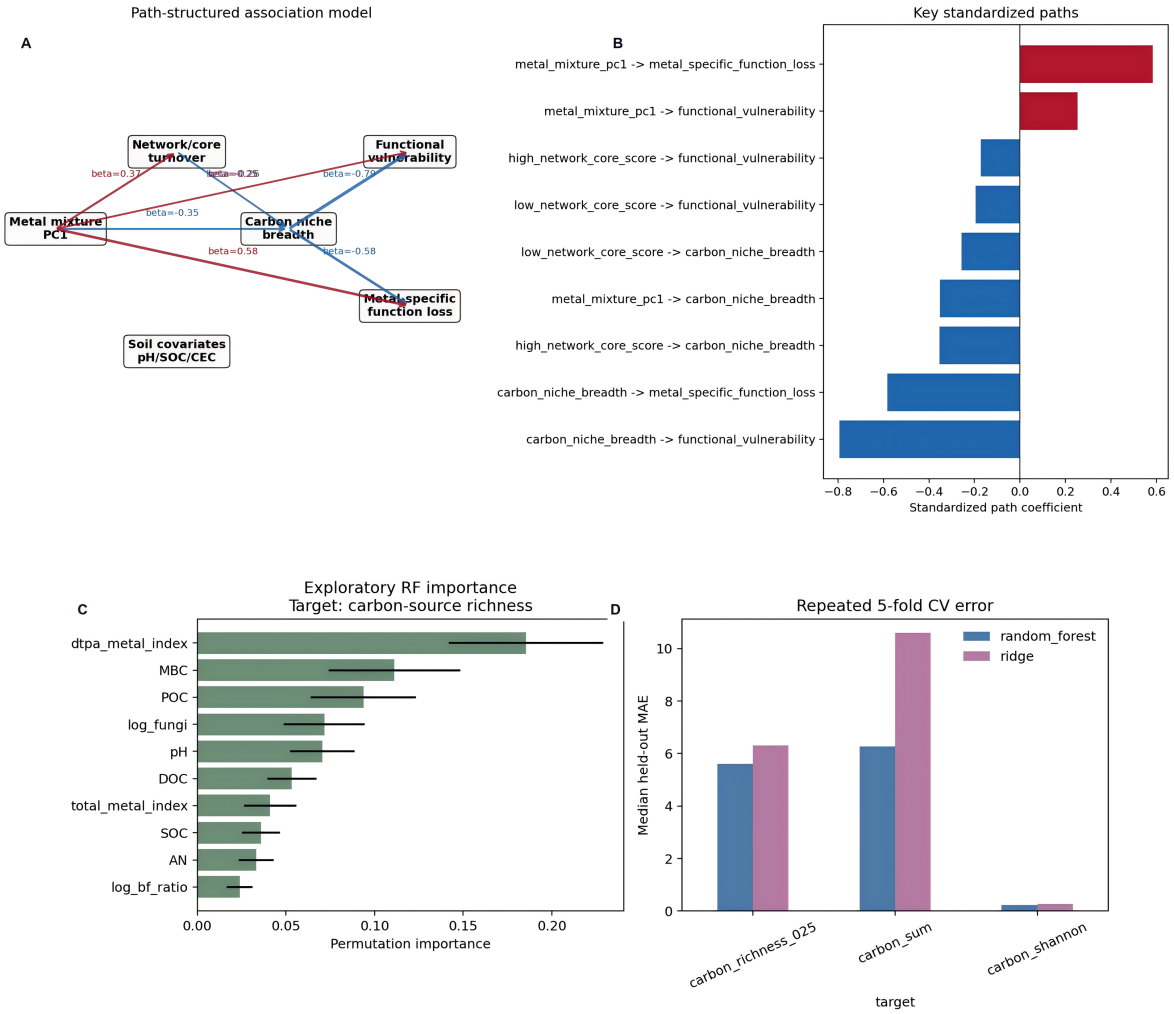
Path-structured model and feature prioritization. **(A)** Path-structured association model linking the DTPA metal-mixture principal component, network-core turnover, carbon-use niche breadth, and functional endpoints; red and blue arrows indicate positive and negative standardized coefficients. **(B)** Standardized path coefficients. **(C)** Random-forest permutation importance for carbon-source richness; bars show mean importance and error bars show variation across repeats. **(D)** Repeated fivefold cross-validation error for random-forest and ridge models. Carbon-use niche breadth formed the strongest functional link between metal pressure and vulnerability.

The path-structured model explained 92.7% of functional-vulnerability variance after integrating metal-mixture pressure, network-core scores, carbon-use niche breadth, pH, SOC, and CEC (Figure 5A). Bioavailable metal-mixture pressure was associated with narrower carbon-use niche breadth (*β* = −0.352, *p* = 0.023), higher functional vulnerability (*β* = 0.254, *p* < 0.001), and higher metal-specific function loss (*β* = 0.583, *p* < 0.001). Carbon-use niche breadth showed the strongest standardized associations with functional vulnerability (*β* = −0.794, *p* < 0.001) and metal-specific function loss (*β* = −0.582, *p* < 0.001).

Bootstrap analysis further identified carbon-use niche breadth as the most stable connecting variable: its indirect associations were significant for both functional vulnerability and metal-specific function loss (bootstrap *p* ≤ 0.005; Figure 5B). Network-core and indicator-taxon pathways were weaker and less stable. This contrast focuses the integrated interpretation on carbon-use niche contraction while retaining community turnover as complementary ecological context.

Random-forest permutation importance independently ranked DTPA metal pressure as the leading feature for carbon-source richness, followed by microbial biomass, particulate organic carbon, fungal abundance, and pH (Figure 5C; Supplementary Figure 4). Repeated cross-validation showed greater error for richness and total carbon use than for Shannon diversity (Figure 5D). Across modeling approaches, carbon-use niche breadth remained the most consistent functional correlate of bioavailable metal pressure.

### Independent contaminated-soil evidence supported the broader ecological context

**Figure 6:**
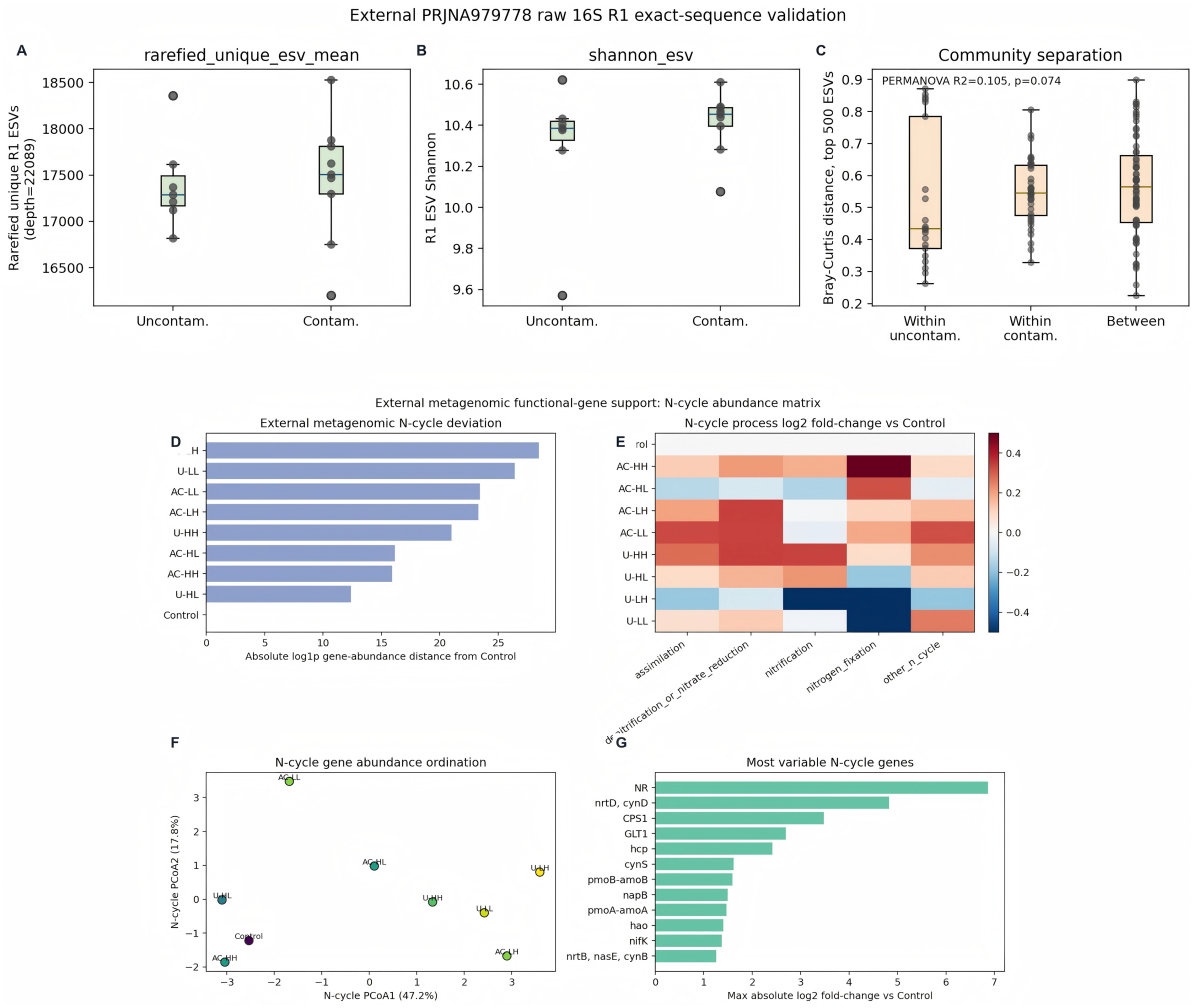
Independent contaminated-soil support for community and C/N functional context. **(A–C)** Exact-sequence richness, Shannon diversity, and Bray-Curtis composition in an independent contaminated agricultural-soil dataset. **(D–G)** Nitrogen-cycling gene profiles and ordination from an independent contaminated-soil metagenomic dataset. Group colors are defined in each panel. The external datasets show community turnover and nutrient-cycle functional shifts across contaminated-soil systems.

Independent contaminated agricultural-soil data showed moderate compositional separation between contaminated and uncontaminated samples despite limited change in richness or Shannon diversity (Figures 6A–C). This pattern parallels the discovery cohort by showing community reorganization without a corresponding collapse in alpha diversity.

Independent metagenomic matrices also showed treatment-related shifts in nitrogen-cycling genes and broader carbon- and nitrogen-functional profiles (Figure 6D–G; Supplementary Figure 5). These datasets extend the field observations across distinct contaminated-soil systems and connect community turnover with altered nutrient-cycle potential.

Supplementary Figures 6 and 7 provide study-design and agricultural exposure-context summaries. These figures frame karst agricultural soils as upstream components of food-production and exposure-prevention systems, without claiming direct evidence for human gut microbiome effects or clinical outcomes.

Microcosm qPCR responses were consistent with the field-derived functional pattern.

**Figure 7:**
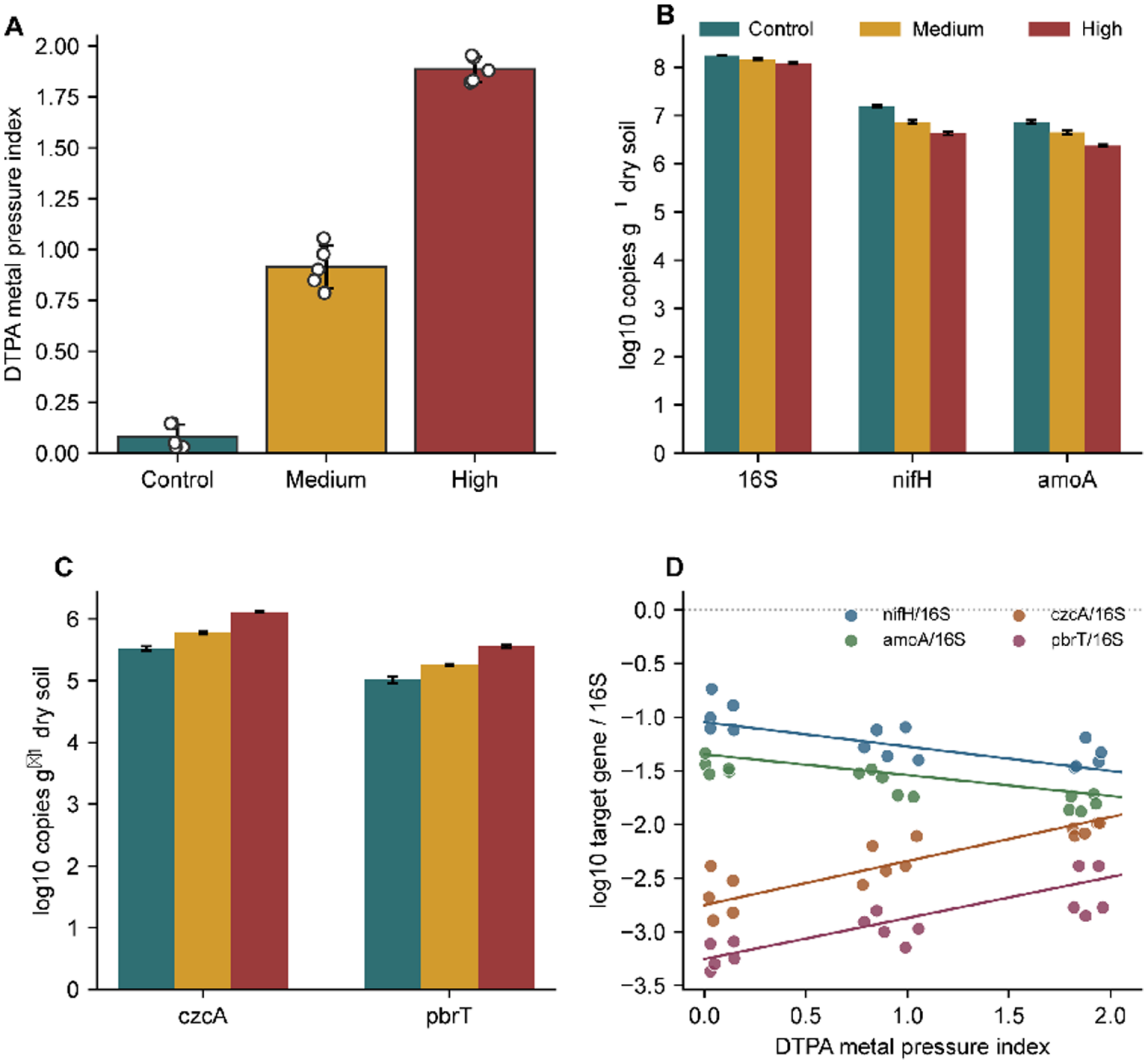
qPCR microcosm responses to metal pressure. **(A)** DTPA-extractable metal-pressure index in control, medium-, and high-metal microcosms. **(B)** Absolute abundance of bacterial 16S rRNA, nifH, and bacterial amoA genes. **(C)** Absolute abundance of czcA and pbrT. **(D)** Associations between DTPA pressure and 16S-normalized functional markers. Points represent biological replicates and bars show mean ± SE. Higher metal pressure enriched resistance markers and reduced nitrogen-cycling markers.

The microcosm experiment established a graded increase in DTPA-extractable metal pressure from the control to medium- and high-metal treatments (Figure 7A). Total bacterial 16S rRNA gene abundance changed modestly across this gradient, whereas functional markers showed stronger treatment-related responses (Figure 7B).

In contrast, nitrogen-cycling markers declined with increasing metal pressure. nifH decreased from 6.97 ± 0.21 to 6.46 ± 0.26 log10 copies g^−1^ dry soil, while bacterial amoA decreased from 6.69 ± 0.15 to 6.08 ± 0.25. The same negative direction was retained after normalization to 16S rRNA gene abundance, indicating that the response was not simply a consequence of total bacterial DNA loss.

The metal-resistance marker czcA increased under high metal pressure, while nifH and bacterial amoA declined in both absolute and 16S-normalized abundance (Figures 7B–D). The opposing responses of resistance and nitrogen-cycling markers demonstrate that the metal gradient altered functional gene allocation without a comparable loss of total bacterial abundance.

## Discussion

This study suggests that bioavailable heavy-metal pressure in karst agricultural soils is associated with a measurable microbial functional-vulnerability phenotype. The central result is not only that metals correlate with microbial endpoints, but that DTPA-extractable metal pressure aligns with carbon-use niche contraction, metal-specific function loss, community reorganization, and path-structured functional vulnerability.

### Bioavailable metals as the relevant microbial exposure axis

DTPA-extractable metals provided a more informative microbial exposure axis than total metal concentrations. In carbonate-rich karst soils, pH, mineral surfaces, organic matter, and cation exchange capacity regulate the mobile and biologically accessible fractions of Pb, Zn, Cu, and Cd. The strong covariance among DTPA-extractable metals further indicates that microorganisms encountered a mixture gradient rather than isolated single-metal exposures. This interpretation is consistent with multi-omics evidence showing that composite metal(loid) pressure reorganizes functional genes involved in carbon, nitrogen, phosphorus, and sulfur cycling (Zhao et al., 2025).

### Carbon-use niche contraction as a functional phenotype

Carbon-use niche contraction was the clearest functional response to the DTPA metal gradient. EcoPlate profiles quantify the breadth, diversity, and relative intensity of substrate use by viable community fractions under standardized assay conditions. They therefore provide a comparative functional phenotype rather than a direct estimate of field carbon flux. The coordinated decline in richness, Shannon diversity, carbon use relative to SOC, and several substrate groups indicates that metal exposure constrained multiple dimensions of carbon acquisition. Similar shifts in community-level physiological profiles have been reported in metal-impacted soils (Senthil Kumar et al., 2023; Liao and Xie, 2007), supporting the ecological relevance of this assay-defined phenotype.

This functional mismatch is especially relevant in karst agricultural soils, where soil fertility, crop residue turnover, and microbial nutrient cycling are tightly linked to land productivity. Broad sensitivity across amino acids, polymers, carbohydrates, and carboxylic acids suggests that bioavailable metal pressure constrains microbial carbon acquisition across multiple resource categories. A global meta-analysis of 155 publications has demonstrated that heavy metal enrichment results in significant decreases in key soil enzyme activities-including 31.9% for dehydrogenase, 24.8% for *β*-glucosidase, 35.8% for invertase, and 24.3% for cellulase-alongside 26.6% inhibition of microbial biomass carbon, providing mechanistic context for the carbon-use contraction observed at the community level (Zeng et al., 2024). Complementary evidence from rice paddy fields indicates that heavy metal contamination reduces microbial carbon use efficiency (CUE) and attenuates the microbial carbon pump, thereby threatening soil carbon sequestration (Xiong et al., 2025). These functional-level observations converge in suggesting that bioavailable metal pressure impairs multiple facets of microbial carbon processing.

### Community reorganization as context, not proof of mechanism

Community ordination, indicator taxa, and network-core turnover converged on the same DTPA-associated gradient. These patterns suggest that functional contraction occurred alongside a redistribution of dominant and highly connected microbial populations. Comparable simplification and reorganization of microbial networks have been observed during succession in metal-contaminated tailings (Li et al., 2025; Wang et al., 2024). In the present dataset, carbon-use niche breadth showed stronger and more stable multivariable associations than network-derived variables, indicating that functional phenotyping captured a more consistent response than any individual community descriptor.

The path-structured association model provides a useful synthesis: bioavailable metal pressure retained an association with vulnerability and function loss, while carbon-use niche breadth served as a stable functional link. Network-core and indicator-taxon scores added community context but did not show stable indirect effects. This suggests that carbon-use niche contraction is the strongest functional phenotype in the current study, whereas network and indicator results should be treated as supportive mechanistic hypotheses.

### Implications for C/N/P cycling and sustainable karst agriculture

The results align with the broader concern that heavy-metal pollution can disrupt essential microbial functions in karst ecosystems. Although the strongest signal emerged from carbon-use phenotypes, the broader relevance to nutrient cycling is supported by independent evidence. A metagenomic study in abandoned Pb–Zn mining areas has demonstrated that Zn and Cd contents are main factors affecting soil carbon and phosphorus cycles during spontaneous succession, with nitrogen-cycling genes reaching their maximum influence during early succession stages (Wang et al., 2024). In karst desertification areas, heavy metals have also been shown to influence soil nitrogen distribution, with effects varying by ecosystem type (Zhang et al., 2023), indicating that metal stress impacts nutrient dynamics in carbonate-derived soils beyond the carbon cycle alone. Reduced microbial carbon-use breadth may influence decomposition, organic matter turnover, nutrient mineralization, and soil recovery after contamination.

The results support a two-dimensional approach to remediation assessment that combines chemical exposure with microbial functional recovery. Total metal concentrations remain necessary for contamination inventories, but they do not resolve the fraction most accessible to microorganisms in carbonate-rich soils. DTPA-extractable metals can therefore complement total concentrations when prioritizing fields, selecting amendments, and monitoring changes in biologically relevant exposure. Carbon-use niche breadth, substrate-group use, and biomass-normalized activity can provide a functional endpoint for determining whether remediation restores microbial carbon-processing capacity. Repeated measurements before and after immobilization, organic amendment, phytoremediation, or land-management interventions would allow chemical improvement to be evaluated together with recovery of soil function.

### One health relevance

Karst agricultural soils are upstream components of food-production and exposure systems. Heavy-metal contamination in these soils may influence crop safety, dietary exposure, and long-term gastrointestinal exposure risks (Tariq et al., 2024). A recent editorial on the global threat posed by metals and metalloids has articulated a One Health approach to mechanisms of toxicity, highlighting that the impact of metals on microbial communities provides insight into their mutual relationships and that soil health is intrinsically linked to human health through food-chain pathways (Lee et al., 2024). The present study does not analyze human gut microbiome samples and does not demonstrate direct gastrointestinal effects. It’s One Health relevance lies instead in exposure prevention and sustainable agriculture: microbial functional vulnerability in contaminated soils may signal broader agroecosystem stress in systems connected to food-chain pathways.

### Limitations

The cross-sectional field design limits temporal and causal inference, and EcoPlate profiles represent standardized carbon-use potential rather than complete *in situ* metabolism. The discovery dataset also lacks shotgun metagenomic, transcriptomic, and metabolomic measurements that could resolve the pathways underlying the observed functional phenotype. The independent 16S and functional datasets broaden the ecological comparison but differ from the discovery cohort in design and analytical resolution. The short-term qPCR microcosm experiment adds controlled evidence for resistance- and nitrogen-cycling marker responses, while longer incubations and direct carbon-flux measurements are needed to connect these responses with field-scale carbon turnover. Future studies should test the proposed indices prospectively across seasons, remediation treatments, and independent karst regions.

Despite these limitations, the study provides a coherent functional-vulnerability framework for interpreting geochemical stress, microbial community reorganization, and carbon-use contraction in contaminated karst agroecosystems.

## Conclusion

Bioavailable heavy-metal pressure in Pb–Zn mining-affected karst agricultural soils was associated with microbial carbon-use niche contraction, metal-specific function loss, community reorganization, and a composite functional-vulnerability phenotype. DTPA-extractable metal mixtures provided a strong exposure axis, while carbon-use niche breadth emerged as the most interpretable functional link between geochemical stress and microbial vulnerability. These findings support a quantitative framework for studying microbial functional disturbance under heavy-metal stress and generate testable hypotheses for carbon-cycle resilience, remediation assessment, and sustainable management of contaminated karst agroecosystems.

## Data Availability

The original contributions presented in the study are included in the article/Supplementary material, further inquiries can be directed to the corresponding authors.

## Supplementary material

SUPPLEMENTARY FIGURE 1 Geochemical risk dominance. This figure summarizes CK-referenced contamination factors, geo-accumulation indices, ecological-risk scores, DTPA/total bioavailability patterns, and explanatory-dominance blocks. The comparison identifies DTPA-extractable multi-metal pressure as the principal microbial exposure axis and relates it to total-metal and ecological-risk metrics.

SUPPLEMENTARY FIGURE 2 Leave-one-group-out robustness. This figure presents leave-one-group-out and sensitivity analyses for the metal-carbon association. The consistent direction across soil-group partitions and winsorized variants demonstrates the robustness of carbon-use niche contraction shown in Figures 2 and 3.

SUPPLEMENTARY FIGURE 3 Phenotype-genotype bridge. This figure links EcoPlate carbon-use phenotypes with carbohydrate-active enzyme (CAZy) profiles. Concordant changes in substrate-use breadth and carbon-processing genes provide functional context for the carbon-use contraction in Figure 3.

SUPPLEMENTARY FIGURE 4 Exploratory machine-learning feature priority. This figure presents random-forest feature prioritization for carbon-source richness. DTPA metal pressure ranked first, followed by microbial biomass, particulate organic carbon, fungal abundance, and pH; repeated cross-validation summarizes model performance across carbon-use endpoints.

SUPPLEMENTARY FIGURE 5 External CAZy and NCyc support. This figure shows CAZy and nitrogen-cycle (NCyc) gene profiles from a long-term Hg-contaminated soil metagenomic dataset. The coordinated carbon- and nitrogen-functional shifts demonstrate broader nutrient-cycle reorganization under metal stress.

SUPPLEMENTARY FIGURE 6 Original study design framework. This figure summarizes the discovery-cohort design, sample groups, geochemical measurements, microbial community variables, and carbon-use functional endpoints integrated in the analysis.

SUPPLEMENTARY FIGURE 7 One Health bridge. This figure maps microbial functional vulnerability in contaminated karst soils onto crop-production, food-chain exposure, and downstream prevention pathways, highlighting the agricultural and One Health relevance of soil functional recovery.
