# Supplementary figures and images for: Bioavailable heavy-metal pressure is linked to microbial functional vulnerability and carbon-use niche contraction in karst agricultural soils
Source: Front Microbiol. 2026 Jul 15;17:1887715. doi: 10.3389/fmicb.2026.1887715 (PMC13416104; doi:10.3389/fmicb.2026.1887715)

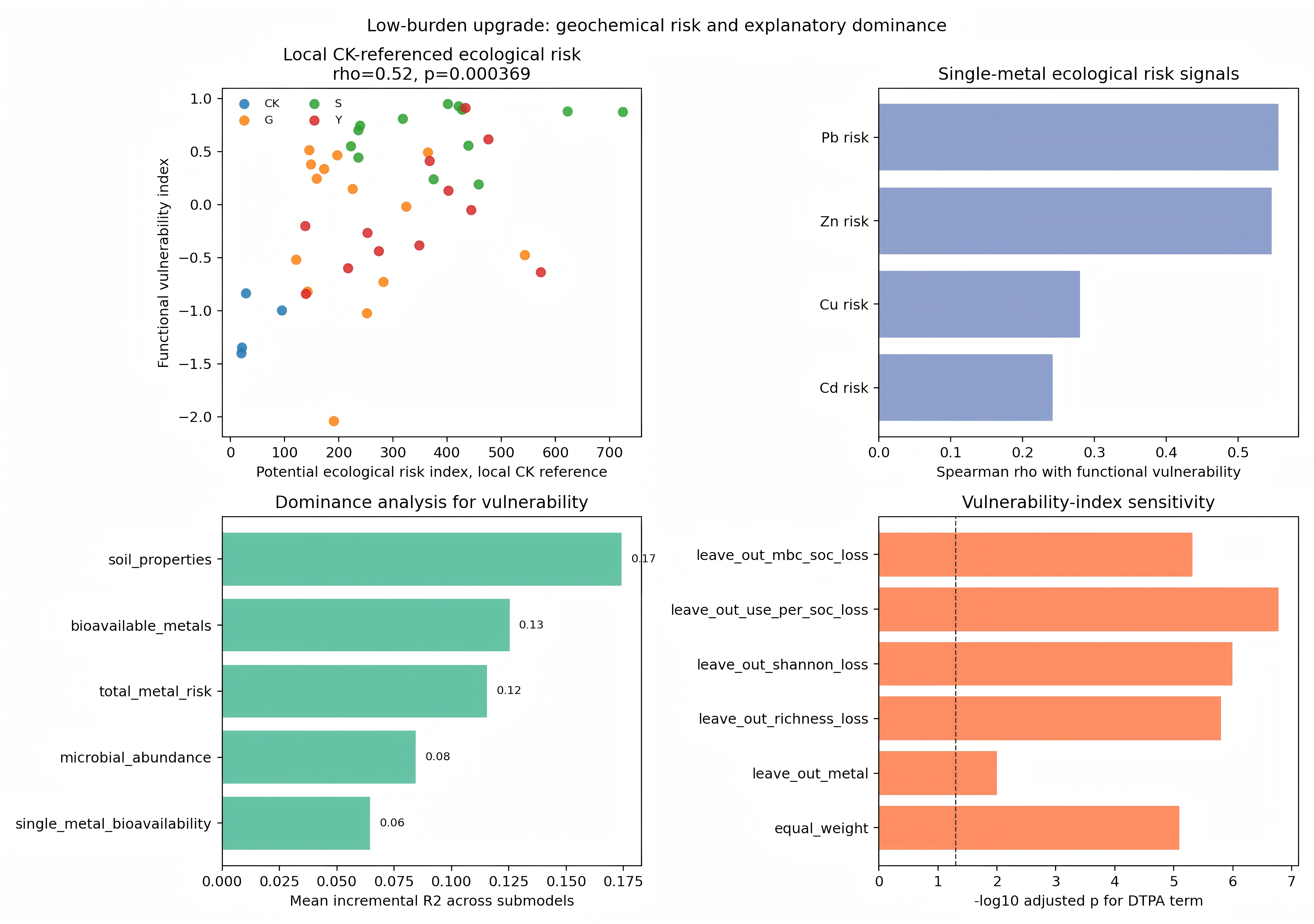

Supplement: SUPPLEMENTARY FIGURE 1 — Geochemical risk dominance. This figure summarizes CK-referenced contamination factors, geo-accumulation indices, ecological-risk scores, DTPA/total bioavailability patterns, and explanatory-dominance blocks. The comparison identifies DTPA-extractable multi-metal pressure as the principal microbial exposure axis and relates it to total-metal and ecological-risk metrics. [file Data_sheet_1.zip › Supplementary/Supplementary_Figure_1_geochemical_risk_dominance.jpg]

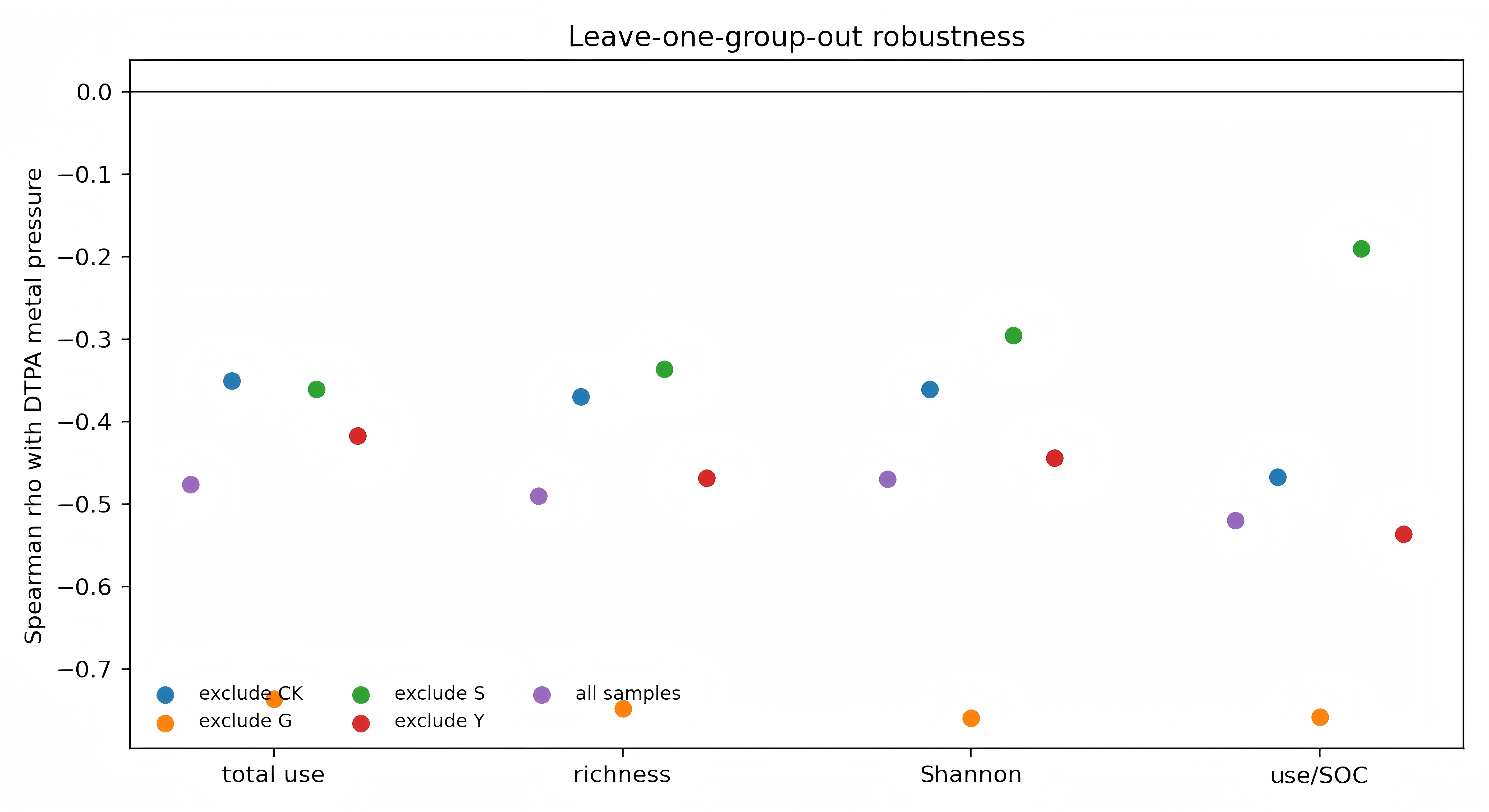

Supplement: SUPPLEMENTARY FIGURE 1 — Geochemical risk dominance. This figure summarizes CK-referenced contamination factors, geo-accumulation indices, ecological-risk scores, DTPA/total bioavailability patterns, and explanatory-dominance blocks. The comparison identifies DTPA-extractable multi-metal pressure as the principal microbial exposure axis and relates it to total-metal and ecological-risk metrics. [file Data_sheet_1.zip › Supplementary/Supplementary_Figure_2_leave_one_group_out_robustness.jpg]

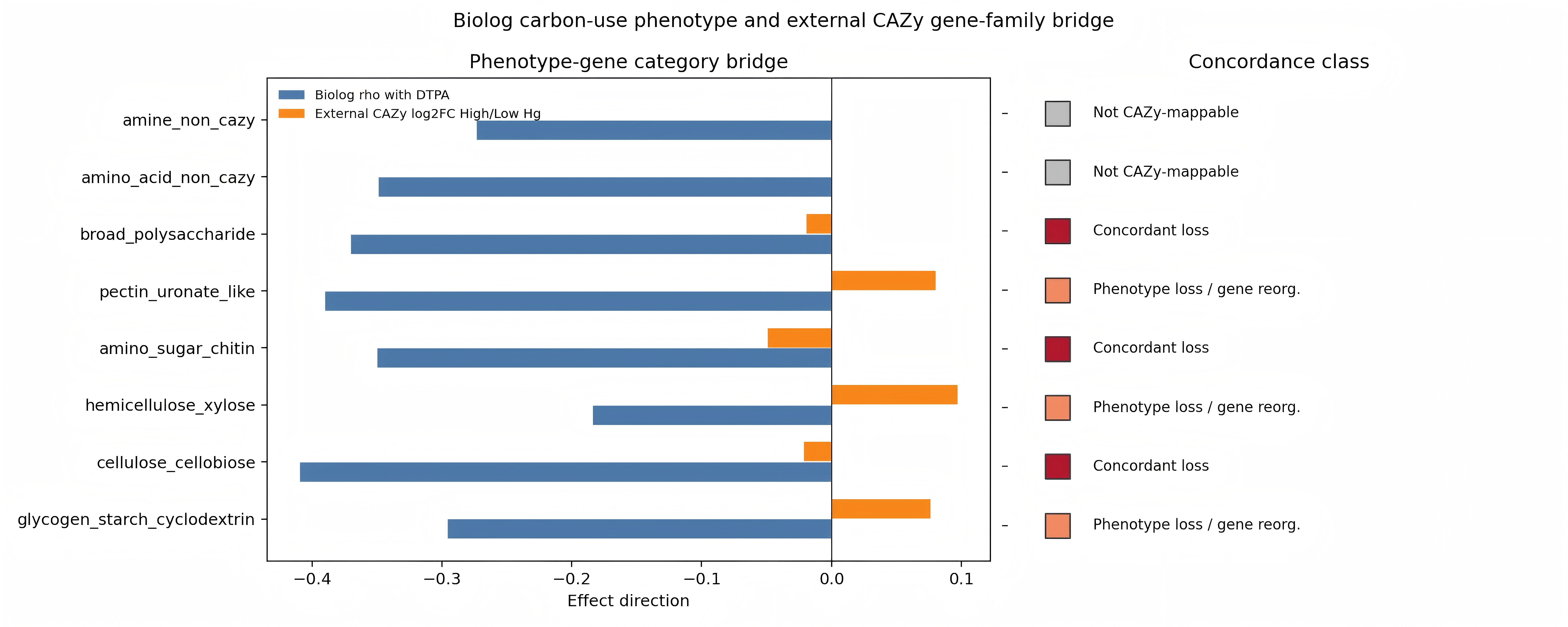

Supplement: SUPPLEMENTARY FIGURE 1 — Geochemical risk dominance. This figure summarizes CK-referenced contamination factors, geo-accumulation indices, ecological-risk scores, DTPA/total bioavailability patterns, and explanatory-dominance blocks. The comparison identifies DTPA-extractable multi-metal pressure as the principal microbial exposure axis and relates it to total-metal and ecological-risk metrics. [file Data_sheet_1.zip › Supplementary/Supplementary_Figure_3_phenotype_genotype_bridge.jpg]

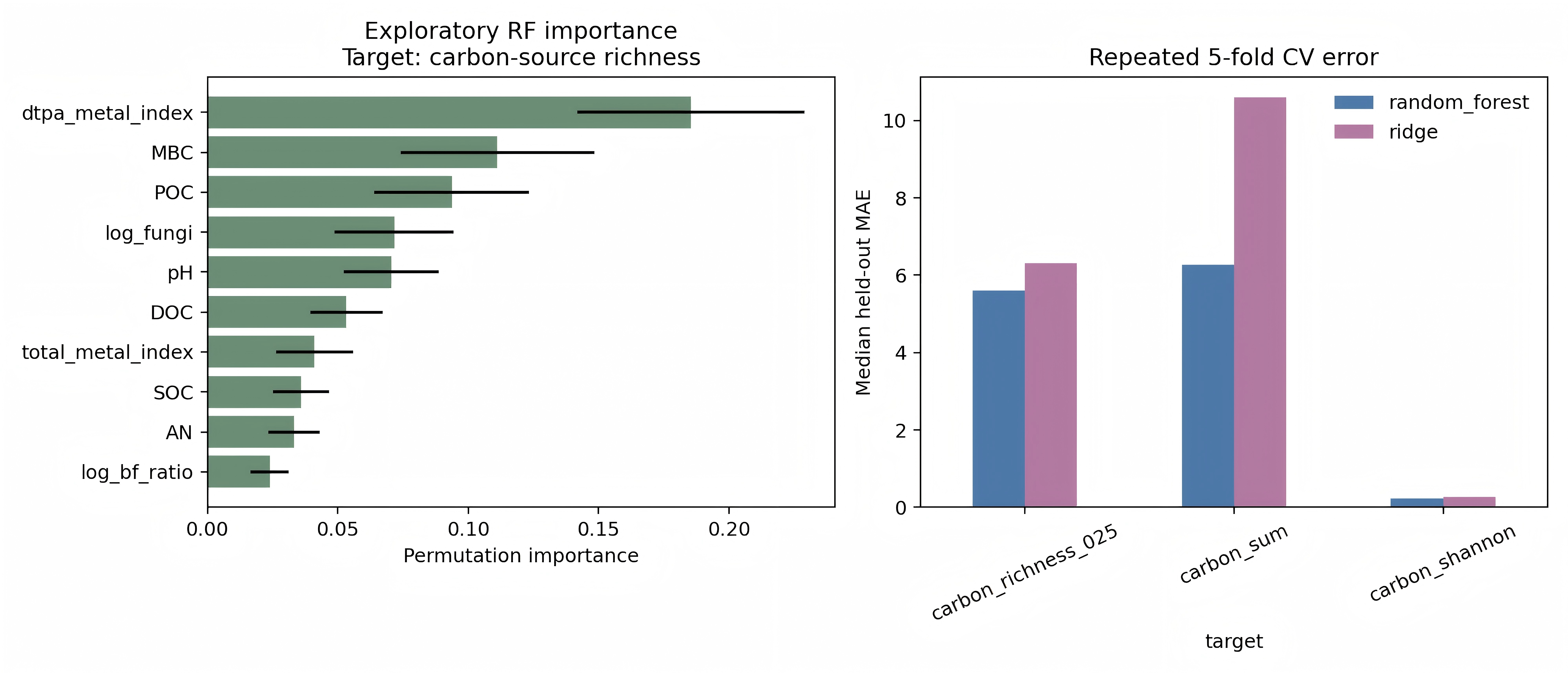

Supplement: SUPPLEMENTARY FIGURE 1 — Geochemical risk dominance. This figure summarizes CK-referenced contamination factors, geo-accumulation indices, ecological-risk scores, DTPA/total bioavailability patterns, and explanatory-dominance blocks. The comparison identifies DTPA-extractable multi-metal pressure as the principal microbial exposure axis and relates it to total-metal and ecological-risk metrics. [file Data_sheet_1.zip › Supplementary/Supplementary_Figure_4_machine_learning_feature_priority.jpg]

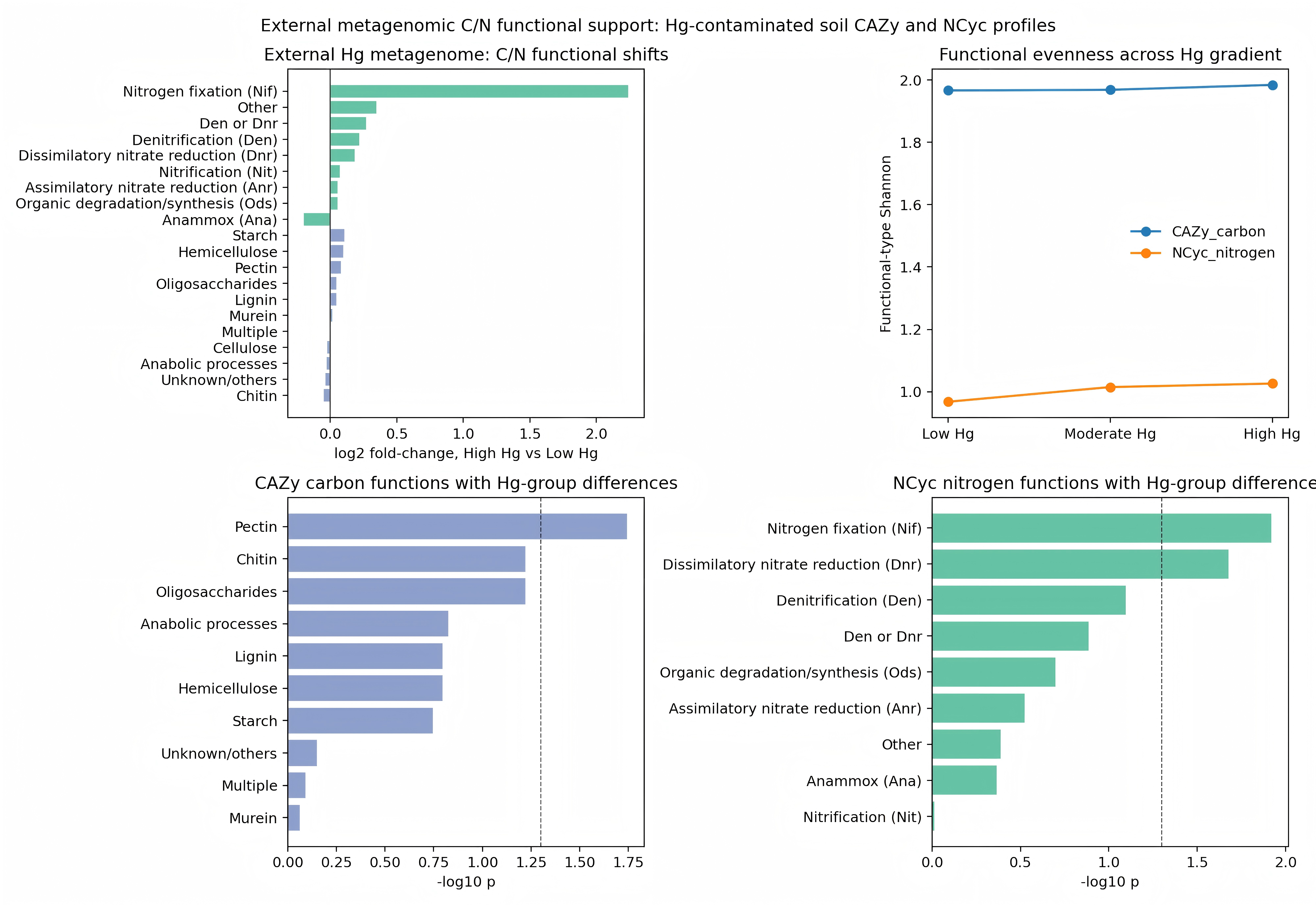

Supplement: SUPPLEMENTARY FIGURE 1 — Geochemical risk dominance. This figure summarizes CK-referenced contamination factors, geo-accumulation indices, ecological-risk scores, DTPA/total bioavailability patterns, and explanatory-dominance blocks. The comparison identifies DTPA-extractable multi-metal pressure as the principal microbial exposure axis and relates it to total-metal and ecological-risk metrics. [file Data_sheet_1.zip › Supplementary/Supplementary_Figure_5_external_cazy_ncyc_support.jpg]

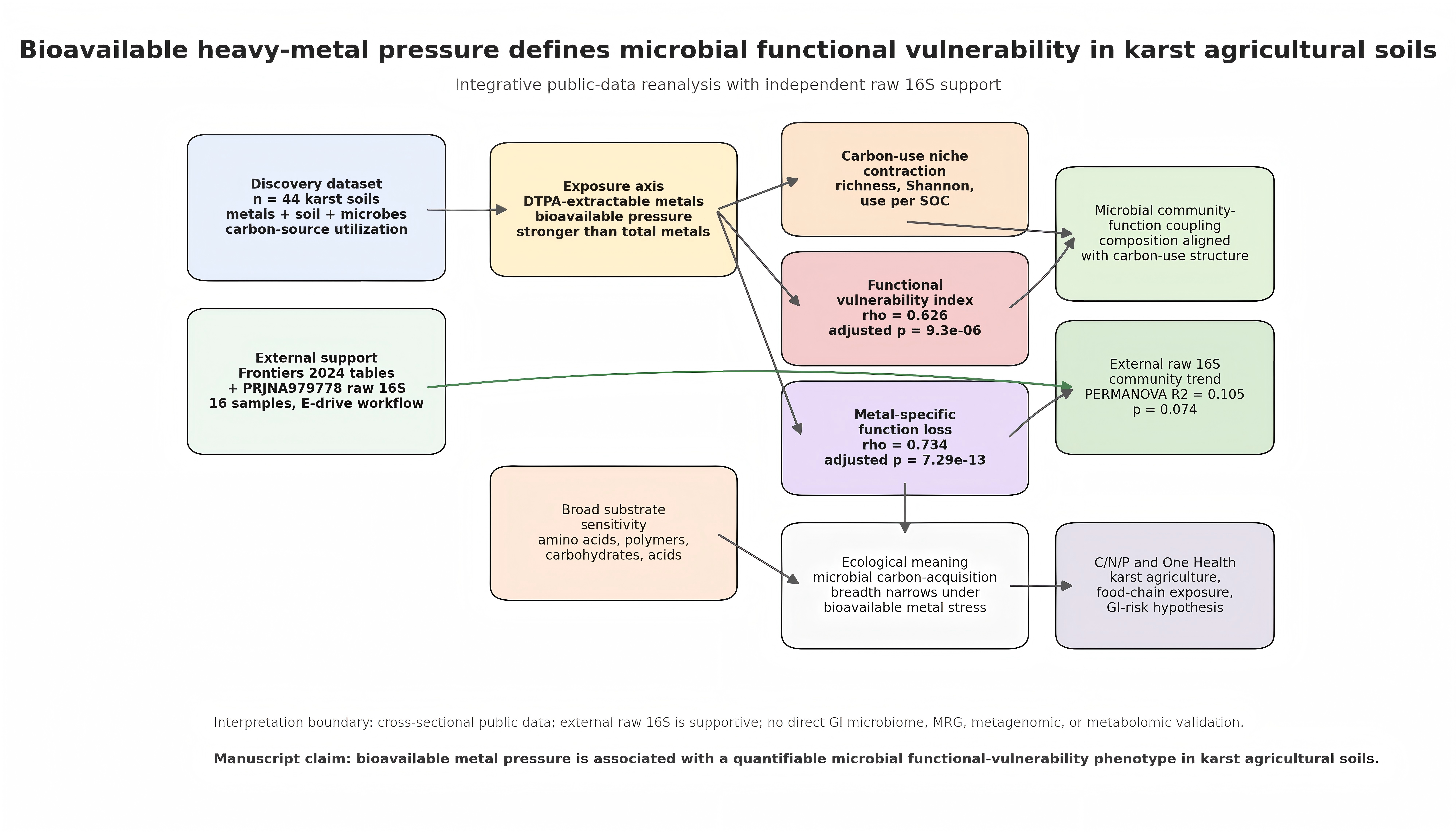

Supplement: SUPPLEMENTARY FIGURE 1 — Geochemical risk dominance. This figure summarizes CK-referenced contamination factors, geo-accumulation indices, ecological-risk scores, DTPA/total bioavailability patterns, and explanatory-dominance blocks. The comparison identifies DTPA-extractable multi-metal pressure as the principal microbial exposure axis and relates it to total-metal and ecological-risk metrics. [file Data_sheet_1.zip › Supplementary/Supplementary_Figure_6_original_study_design_framework.jpg]

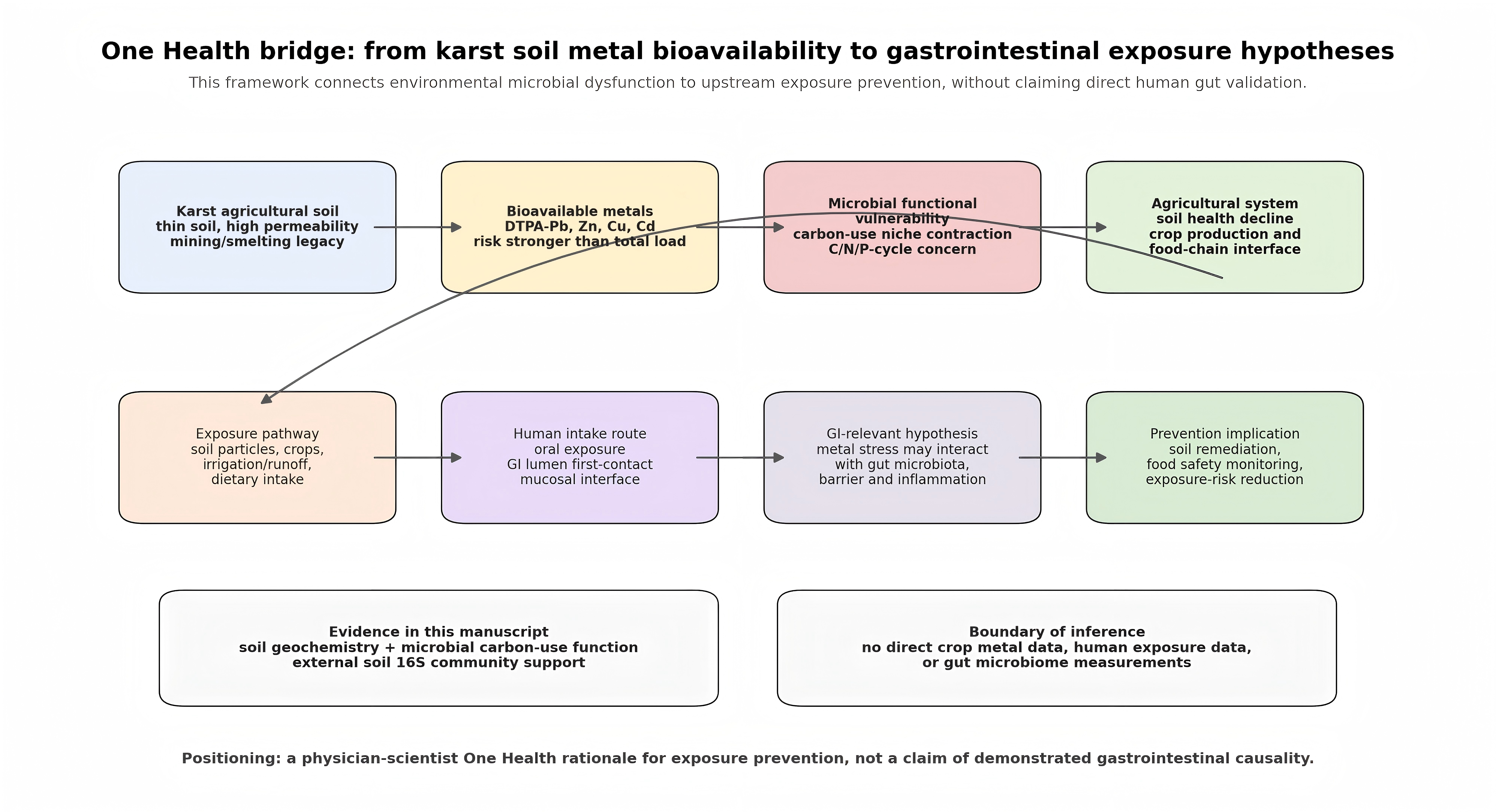

Supplement: SUPPLEMENTARY FIGURE 1 — Geochemical risk dominance. This figure summarizes CK-referenced contamination factors, geo-accumulation indices, ecological-risk scores, DTPA/total bioavailability patterns, and explanatory-dominance blocks. The comparison identifies DTPA-extractable multi-metal pressure as the principal microbial exposure axis and relates it to total-metal and ecological-risk metrics. [file Data_sheet_1.zip › Supplementary/Supplementary_Figure_7_one_health_bridge.jpg]
